# A microorganisms’ journey between plant generations

**DOI:** 10.1186/s40168-018-0459-7

**Published:** 2018-04-26

**Authors:** Nathan Vannier, Cendrine Mony, Anne-Kristel Bittebiere, Sophie Michon-Coudouel, Marine Biget, Philippe Vandenkoornhuyse

**Affiliations:** 1 Université de Rennes 1, CNRS, UMR 6553 EcoBio, campus Beaulieu, Avenue du Général Leclerc, 35042 Rennes Cedex, France; 2 Université de Lyon 1, CNRS, UMR 5023 LEHNA, 43 Boulevard du 11 Novembre 1918, 69622 Villeurbanne Cedex, France; 3 Université de Rennes 1, CNRS, UMS3343 OSUR, campus Beaulieu, Avenue du Général Leclerc, 35042 Rennes Cedex, France

**Keywords:** Clonal plants, Microbiota, 16S/18SrRNA, Vertical transmission, Microorganisms dispersal

## Abstract

**Background:**

Plants are colonized by a great diversity of microorganisms which form a microbiota and perform additional functions for their host. This microbiota can thus be considered a toolbox enabling plants to buffer local environmental changes, with a positive influence on plant fitness. In this context, the transmission of the microbiota to the progeny represent a way to ensure the presence of beneficial symbionts within the habitat. Examples of such transmission have been mainly described for seed transmission and concern a few pathogenic microorganisms. We investigated the transmission of symbiotic partners to plant progeny within clonal plant network.

**Methods:**

We used the clonal plant *Glechoma hederacea* as plant model and forced newly emitted clonal progeny to root in separated pots while controlling the presence of microorganisms. We used an amplicon sequencing approach of 16S and 18S rRNA targeting bacteria/archaea and fungi respectively to describe the root microbiota of mother and clonal-plant offspring.

**Results:**

We demonstrated the vertical transmission of a significant proportion of the mother plants’ symbiotic bacteria and fungi to the daughters. Interestingly, archaea were not transmitted to the daughter plants. Transmitted communities had lower richness, suggesting a filtration during transmission. We found that the transmitted pool of microorganisms was similar among daughters, constituting the heritability of a specific cohort of microorganisms, opening a new understanding of the plant holobiont. We also found significant effects of distance to the mother plant and of growth time on the richness of the microbiota transmitted.

**Conclusions:**

In this clonal plant, microorganisms are transmitted between individuals through connections, thereby ensuring the availability of microbe partners for the newborn plants as well as the dispersion between hosts for the microorganisms. This previously undescribed ecological process allows the dispersal of microorganisms in space and across plant generations. As the vast majority of plants are clonal, this process might be therefore a strong driver of ecosystem functioning and assembly of plant and microorganism communities in a wide range of ecosystems.

**Electronic supplementary material:**

The online version of this article (10.1186/s40168-018-0459-7) contains supplementary material, which is available to authorized users.

## Background

All living plants experience interactions with endophytic microorganisms and are known to harbor a great diversity of symbionts (i.e., long-lasting interactions) including fungi [1, 2], bacteria [3–5], and archaea [6] which collectively form the plant microbiota. This microbiota performs ecological functions that extend the plant’s ability to adapt to environmental conditions [7, 8]. Studies using maize cultivars demonstrated that genetic control of the composition of the microbial rhizosphere by the host plant was detectable, even if limited [9]. Plant microbiota composition is thus, at least in part, not only a consequence of the pool of microorganisms available for recruitment in the surrounding soil but also of plant selective recruitment within the endosphere. This filtering system includes plant defense and plant-microbe signaling mechanisms [10–12], as well as promotion of the best cooperators through a nutrient embargo toward less beneficial fungi [8, 13].

From a theoretical point of view, vertical and pseudo-vertical transmissions (i.e., inheritance of conspecific symbionts from parents to offspring sharing the same environment) [14] are advantageous because they limit the costs of foraging for suitable symbionts [15]. Vertical transmission would thus permit a “continuity of partnership” between the plant and its symbionts [16]. In this context, microbiota heritability is also a way for the plant to ensure environmental quality for its progeny. *In natura*, plants can reproduce either by seed production or by clonal multiplication [17, 18]. Some studies have evidenced a vertical inheritance of endophytic symbionts colonizing host plants through the seeds: the most well-known example is perhaps the transmission of the stress-protective endophyte *Neotyphodium coenocephalum* to the descendants in several grass plant species [19, 20].

Recent findings suggest that the vegetative elongation of the horizontal stems forming the clonal plant network is accompanied by the transmission of a “cohort” of microorganisms that includes arbuscular mycorrhizal fungi, to spatially distant clonal offspring [21]. This form of heritability of microorganisms to plant offspring is not mediated environmentally (i.e., through environment sharing) or sexually. Such process would support the niche construction of clonal plant offspring while microorganisms could benefit from a selective dispersal vector allowing them to reach a similar and hence suitable host. Transmission in clonal plants has been demonstrated to involve information- and resource-sharing within the physical clone (i.e., physiological integration) [22]. An additional level of integration might occur through the sharing of microorganisms within the clonal network, as previously proposed by Stuefer et al. [23].

**Fig. 1:**
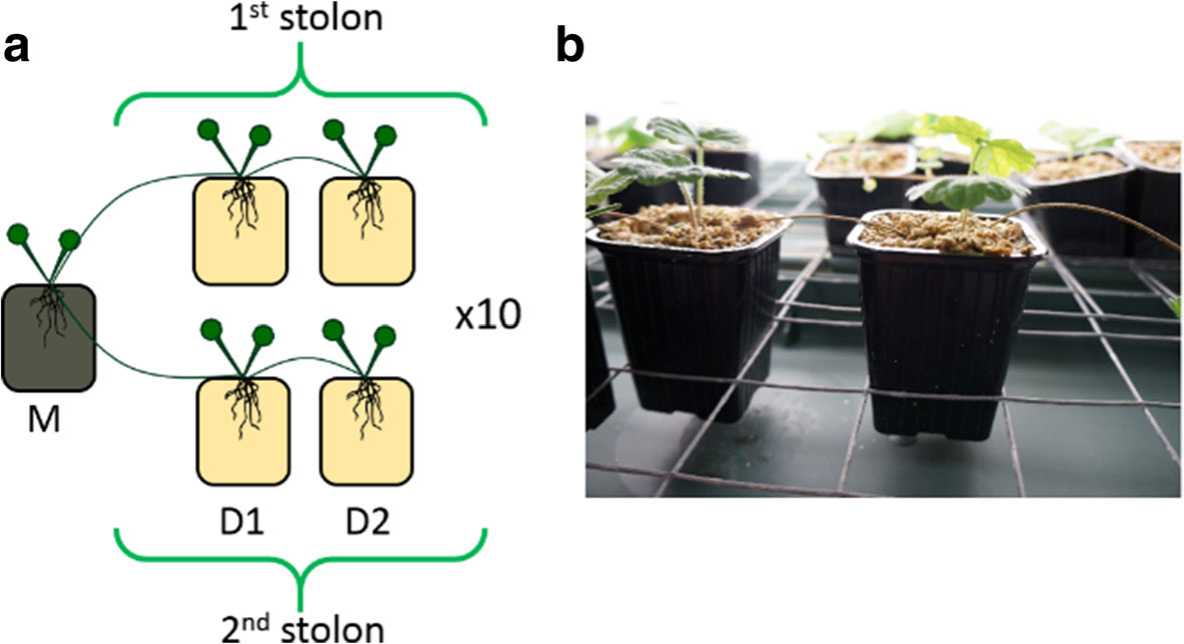
Experimental design. **a** Clonal ramets of 10 ecotypes were forced to root in separate individual pots and connected by stolons. At the end of the experiment, the clonal network consisted of the mother ramet and four daughter ramets. The daughter ramets (1st and 2nd mother ramets) were positioned along the two primary stolons produced by the mother ramet. Pots with mother ramets were filled with homogenized field soil, those with daughter ramets contained sterilized substrate, and contact was only by the internode that separated two consecutive ramets. M mother, D1 1st daughter, D2 2nd daughter. **b** Picture of the experimental design: the pots are only connected by the internodes

We tested the hypothesis of microorganism transmission to clonal offspring through clonal integration and addressed the new concept of a shared microbiota heritability in clonal plants, using the clonal herbaceous species *Glechoma hederacea* as model. The growth form of this plant consists of a network of ramets connected through horizontal stems (i.e., aerial stolons), one of the most widespread forms of clonality [16]. The daughter ramets produced can be separated from the mother ramet after a disturbance or by the natural senescence of the physical connections between ramets during the growth of the clonal network and are then able to grow and reproduce sexually on their own. Plants from 10 ecotypes were grown under controlled conditions. First, a juvenile ramet without roots (mother ramet) was transplanted into a pot containing field soil. Plant growth was oriented by forcing the newly emitted ramets (daughter ramets) of the two ramifications to root into separate pots containing sterilized substrate (Fig. 1). Our aim was to detect the endophytic microorganisms present in the mother ramet roots and transferred to the daughter ramets through the clone stolons. High-throughput amplicon sequencing of 16S and 18S rRNA genes was used to detect and identify bacteria, archaea, and fungi within the root endosphere and the stolon internodes. Control pots randomly distributed in the experiment were also analyzed to remove from the dataset all operational taxonomic units (OTUs) which could not be attributed to a plant-mediated transfer of microorganisms (see methods in Additional file 1).

## Methods

### Biological material

We used the clonal, perennial herb *Glechoma hederacea*, which is a common model for studying clonal plant response to environmental constraints [24–26]. *G. hederacea* clones produce new erect shoots at the nodes at regular intervals of 5 to 10 cm (the internodes) on plagiotropic monopodial stolons (i.e., aboveground connections). Each ramet consists of a node with two leaves, a root system, and two axillary buds. In climatic chambers with controlled conditions and in the absence of enriched substrate, *G. hederacea* does not invest in flowering but displays only vegetative growth [25]. The ramets used in our experiments were obtained from the vegetative multiplication of 10 clonal fragments taken at 10 different locations separated by at least 1 km to sample different ecotypes. Plants were grown for 3 months with a diurnal cycle of 12 h day/12 h night at 20 °C on a vermiculite substrate to limit parental effects related to their geographic location and habitats [27]. Vegetative multiplication was carried out on a sterilized substrate (50% sand and 50% vermiculite, autoclaved twice at 120 °C for 1 h).

### Experimental conditions

Experiments were carried out with cultures grown on the same sterile substrate (50% sand, 50% vermiculite) in a climate-controlled chamber with a diurnal cycle of 12 h day/12 h night at 20 °C. Plants were watered with deionized water every 2 days to ensure moisture. Necessary nutrients were supplied by watering the plants every 10 days with a low-phosphorus watering solution to favor mycorrhization [22]. At each watering, the volumes of deionized water and fertilizing solution per pot were 25 mL. To test for the transmission of microorganisms within the clonal network, we transplanted an initial ramet (mother ramet) into a pot with field soil and oriented its growth to force the newly emitted ramets to root in different individual pots containing sterilized substrate (Fig. 1). We used 10 clonal fragments in total corresponding to the 10 abovementioned ecotypes. Each of them produced a clonal network comprising 5 ramets (1 mother ramet and 4 daughter ramets) (e.g., 50 root samples). The composition of microbiota is analyzed at the mother ramet level and compared to the daughter ramets within each ecotype. This design based on 10 replicates, each of them corresponding to a different ecotype, enables to take into account the natural variability of clonal fragment responses to the tested soil composition. It ensures that the results observed is not due to a particular ecotype but can be considered as a general pattern of *Glechoma hederacea* species. During the experiment, secondary ramifications of daughter ramets were removed to limit spread and confine the growth of the plant to a simple network of five ramets comprising the mother ramet and four daughter ramets equally distributed between two stolons (two on each primary stolon). By using two stolons, we could test whether the potential transmission was systematic within the clone or whether this transmission varied between stolons (i.e., transfer of random organisms from the mother pool). The transplanted clonal unit (i.e., the mother ramet) consisted of a mature ramet (leaves and axillary buds) with one connective stolon internode (to provide resources to support ramet survival) [28], and without roots (to avoid prior colonization of the roots by micro-organisms). Soil has been collected in a frequently mown mesic grassland close to the interface with the hedgerows where *Glechoma hederacea* populations were developing. Soil is typically cambisols with a soil parent material of schist. Aboveground floristic composition comprises between 5 and 10 different plant species with *L. perenne* and *T. repens* as the most abundant one. Soil was then sieved through 0.5 cm mesh to remove stones and roots. The experiment was stopped and the ramets harvested when the clone had reached the stage with a mother ramet and four rooted daughter ramets. The composition of endospheric microorganisms in the root and internode samples was analyzed by separating the clonal network into stolon internodes, roots, and shoots for both the mother and the daughter ramets. Each internode and root sample was meticulously washed first with water, secondly with a 1% Triton × 100 (Sigma) solution (three times) and lastly with sterile water (five times). This procedure ensured removal of non-endospheric microorganisms [29]. In order to control for potential contaminations, three control pots were randomized into the experimental design. These pots were filled with the same sterile substrate and watered similarly to the other pots. Substrate from these control pots was sampled at the end of the experiment so that all contaminant microorganisms that were not plant transmitted could be removed from the sequence analyses and from all subsequent statistical analyses. All root, internode, and substrate samples were frozen at − 20 °C before DNA extraction and subsequent molecular work.

### DNA extraction and amplification

DNA was extracted from cleaned roots and internodes, as well as from the substrate from control pots, using the DNeasy plant mini kit (Qiagen). The 18S rRNA gene was PCR amplified using fungal primers NS22b (5′-AATTAAGCAGACAAATCACT-3′) and SSU817 (5′-TTAGCATGGAATAATRRAATAGGA-3′) [2]. The conditions for this PCR comprised an initial denaturation step at 95 °C for 4 min followed by 35 cycles of 95 °C for 30 s, 54 °C for 30 s, and 72 °C for 1 min with a final extension step at 72 °C for 7 min. The 16S rRNA gene was amplified using bacterial primers 799F (5′-AACMGGATTAGATACCCKG-3′) and 1223R (5′-CCATTGTAGTACGTGTGTA-3′). The conditions for this PCR consisted of an initial denaturation step at 94 °C for 4 min followed by 32 cycles of 94 °C for 30 s, 54 °C for 30 s, and 72 °C for 1 min with a final extension step at 72 °C for 10 min. The 16S rRNA gene was also amplified using a nested PCR with archaea primers. The first PCR primers were Wo_17F (5′-ATTCYGGTTGATCCYGSCGRG-3′) and Ar_958R (5′-YCCGGCGTTGAMTCCAATT-3′) and PCR conditions comprised an initial denaturation step at 94 °C for 2 min followed by 35 cycles of 94 °C for 30 s, 57.5 °C for 50 s, and 72 °C for 50 s with a final extension step at 72 °C for 10 min. The second PCR primers were Ar_109F (5′-ACKGCTCAGTAACACGT-3′) and Ar_915R (5′-GTGCTCCCCCGCCAATTCCT-3′) and PCR conditions comprised an initial denaturation step at 94 °C for 4 min followed by 32 cycles of 94 °C for 30 s, 57 °C for 30 s, and 72 °C for 1 min with a final extension step at 72 °C for 10 min. All amplification reactions were prepared using Illumina RTG PCR beads (GE Healthcare) with 2 μL of extracted DNA and target PCR products were visualized by agarose gel electrophoresis.

### Sequencing and data trimming

All PCR amplification products were purified using Agencourt AMPure XP kit. After purification, the amplification products were quantified and their qualities checked using Agilent high sensitivity DNA chip for BioAnalyzer (Agilent) and Invitrogen fluorimetric quantification (Quant-iT PicoGreen dsDNA Assay Kit, ThermoFisher Scientific). Quality was estimated by the size of the amplicons produced regarding the expected size, the absence of primers dimers, and the molarity and concentration of the PCR products.

All PCR amplifications products were then subjected to an end repair step and adaptor ligation using the NEBNext Ultra II DNA Library Prep Kit for Illumina (New England Biolabs). Multiplexing was done with a PCR step using NEB next Ultra 2 multiplex oligo (dual index, New England Biolabs). Multiplexed products were then quantified and quality checked using Agilent high sensitivity DNA chip for bioanalyzer and quantitative PCR with SmartChip martchip RT PCR (Takara-Wafergen). Amplicons libraries were pooled to equimolar concentration, a quantitative PCR using LightCycler 480 SYBR Green I Master (Roche) was performed and products were paired-end sequenced (2 × 250 bp) with an Illumina MiSeq instrument at the Human and Environmental Genomic Plateforme of Rennes (France). Data trimming consisted of different steps: primer removal (Cutadapt) and suppression of sequences containing unidentified bases. An additional step consisted of checking the sequence orientation using a homemade script. This stringent data trimming resulted in 9,592,312 reads. Trimmed sequences were then analyzed using the FROGS pipeline [30] (bio-informatic workbench “X.SIGENAE” [http://www.sigenae.org/]). FROGS pre-process was performed with a custom protocol [31] for archaea and fungi and with the FROGS standard protocol for bacteria reads. In this pre-process, bacteria reads were assembled using Flash [32]. The clustering step was performed with SWARM to avoid the use of identity thresholds to group sequences in OTUs [33]. Following the pipeline designer’s recommendations, a de-noising step was performed with a maximum distance of aggregation of 1 followed by a second step with a maximum distance of aggregation of 3. Chimera were filtered with the FROGS remove chimera tool. A filter was also applied to keep those OTUs with sequences in at least three samples to avoid the presence of artificial OTUs. All statistical analyses were also done with a five samples filter and results were similar. We herein present only the R2 fungi and R1 archaea results based on affiliation statistics that indicated a better quality of affiliation. OTUs affiliation was performed using Silva 123 16S for bacteria and archaea and Silva 123 18S for fungi. OTUs were then filtered based on the quality of the affiliations with a threshold of at least 95% coverage and 95% BLAST identity. The stringent parameters used in FROGS enabled us to finally obtain 4,068,634 bacterial reads, 2,222,950 fungal reads, and 113,008 archaeal reads. Rarefaction curves were generated using R (version 3.3.0) with the function “rarefaction” in the package vegan (2.2–1) [34]. We produced mean rarefaction curves for bacterial fungal and archaeal communities for roots, stolons, and control pot samples to determine whether the sequencing depth was sufficient to describe the expected number of operational taxonomic units (OTUs). The sequencing depth was high enough to describe the microbial communities in detail (Additional file 1: Figure S1). To homogenize the number of reads by sample for subsequent statistical analyses, samples were normalized to the same number of reads based on graphical observation of the rarefaction curves using the same R package. During this step, samples with less reads than the normalization value were removed from the dataset. All OTUs found in the soil of the control pots were then removed from the data set. The three control pots contained 0 archaeal reads. Two out of the three control pots contained 0 fungal reads and we found a total of 3371 fungal reads distributed in 19 OTUs in the last pot. The three control pots also contained 65,378, 33,773, and 37,587 bacterial reads distributed respectively in 153, 313, and 219 OTUs.

Sequences data are available through the accession number PRJEB20603 at European Nucleotide Archive. Fungal, bacterial and archaeal processed datasets are also available as additional materials (Additional files 2, 3 and 4).

### Statistical analyses

The positions and stolon of each ramet within the network were recorded as two factors for the statistical analyses. We considered three positions in the network: the mother ramet, the 1st daughter ramet and the 2nd daughter ramet. The stolon was considered as a factor with two levels: the 1st and the 2nd stolon emitted during growth. We analyzed heritability, richness and composition of microorganism assemblages in *G. hederacea* ecotypes. We analyzed fungi and bacteria assemblages separately. No statistical analyses were performed on archaea data as they were found in the mother ramet roots and in the stolon internodes following the mother ramets but not in the daughter roots. All statistical analyses were performed using the R software [35].

#### Heritability calculation and null model construction

Heritability was measured for each taxonomic group in each ecotype as the number of OTUs present in the mother ramet and shared by at least two daughter ramets (we also tested the heritability calculation for three and four daughter ramets). To determine whether the observed heritability could be expected stochastically, we compared the observed heritability against a null model. This procedure is designed to test the null hypothesis that species from the mother ramets are randomly distributed within each daughter ramet and do not reflect the selection or the dispersal of a particular set of species from the mother pool. It allows assessment of the probability that the observed heritability indexes are greater than would be expected under a null distribution [36]. We built a null model for each of the 10 ecotypes by generating daughter ramet communities with a random sampling of microorganism species within the mother’s pool. The probability of species sampling was the same for all species in the mother’s pool (i.e., independent of their initial abundance in the mother roots). Only species identity was changed from one model to another while species richness within the daughter communities remained unmodified. For each daughter ramet community within the 10 ecotypes, 9999 virtual communities were randomly sampled from the mother’s pool and the heritability indexes calculated for each of these models. Results were similar when a less stringent heritability was used (e.g., OTU present in at least one daughter ramet) but the heritability could not be more stringent because it would create null communities with zero inherited OTUs for most of the null communities and thus overestimate the difference between the observed and the random heritability values.

For each ecotype, we computed the standard effect size (SES), calculated as described by Gotelli and McCabe [37]:

$$ \mathrm{SES}=\frac{I_{\mathrm{obs}}-{I}_{\mathrm{null}}}{\sigma_{\mathrm{null}}} $$

where *I*_obs_ is the observed heritability index value, *I*_null_ is the mean of the null distribution, and *σ*_null_ is its standard deviation. SES aims to quantify the direction and magnitude of each ecotype heritability index compared to the null distribution. Negative SES values indicate lower heritability than in the random model (heritability of microorganisms species not present in the mother ramet), whereas positive SES values reveal higher heritability than expected by random (heritability of microorganisms from the mother ramet). A one-sample *t* test with the alternative hypothesis “greater” was then applied to the SES values to determine whether they were significantly greater than zero after checking for the data normality.

#### Analyses of richness through linear mixed models

Richness was calculated as the number of OTUs present in the sample. Richness was calculated separately for bacteria and fungi at the scale of the whole community and at the scale of the phyla (OTU richness in each phylum). We chose these two scales to detect general patterns in microorganism richness and also to detect potential variation in these patterns between taxonomic groups (phyla). We conducted our analyses at the phylum scale rather than at a more precise taxonomic level because we were constrained by the sequence affiliation that produced multi-affiliation of OTUs at lower taxonomic levels. To test whether the richness was affected by the sample position in the clonal network, we performed linear mixed-effects models using R packages “nlme” [38] and “car” [39] with functions “lme” and “anova.” We initially tested for differences in richness between mother and daughter ramets. We then tested for differences in richness between 1st and 2nd daughter ramets by considering the position in the clone (1st daughter or 2nd daughter) and the stolon (1st stolon, 2nd stolon) within the plant ecotype as explanatory variables. Ecotype-induced variance and statistical dependency were controlled by considering the position in the clone (mother or daughter) and the stolon as fixed factor and the plant ecotype as a random factor in the mixed models. Normality of the models residuals was verified using a graphical representation of the residuals and the data were log or square root transformed when necessary. For several fungal and bacterial groups exhibiting low abundances in the samples, the models testing differences in richness did not ensure the normality of the residuals and thus these results are not presented.

#### Analyses of microorganisms community composition

A PLS-DA (partial least square discriminant analysis) analysis was used to test whether the microbiota composition varied significantly between mother and daughter ramets and between daughter ramets. The PLS-DA consists of a partial least squares (PLS) regression analysis where the response variable is categorical (y-block; describing the position in the ecotype), expressing the class membership of the statistical units [40–42]. This procedure makes it possible to determine whether the variance of the x-blocks can be significantly explained by the y-block. The x-blocks (OTUs abundance) are pre-processed in the PLS-DA analysis using an autoscale algorithm (i.e., centers columns to zero mean and scales to unit variance). The PLS-DA procedure includes a cross-validation step producing a *p* value that expresses the validity of the PLS-DA method regarding the data set. The PLS-DA procedure also expresses the statistical sensitivity indicating the modeling efficiency in the form of the percentage of misclassification of samples in categories accepted by the class model. Our aim in using this model was to test the variance of community composition that could be explained by the position of the ramet in the clone. The entire data set was subdivided into two or three groups depending on the groups tested (i.e., mother ramets vs 1st daughter ramets vs 2nd daughter ramets, mother ramets vs all daughter ramets and 1st daughter ramets vs 2nd daughter ramets).

## Results

### Archaeal, bacterial, and fungal communities in the roots of *Glechoma hederacea*

**Fig. 2:**
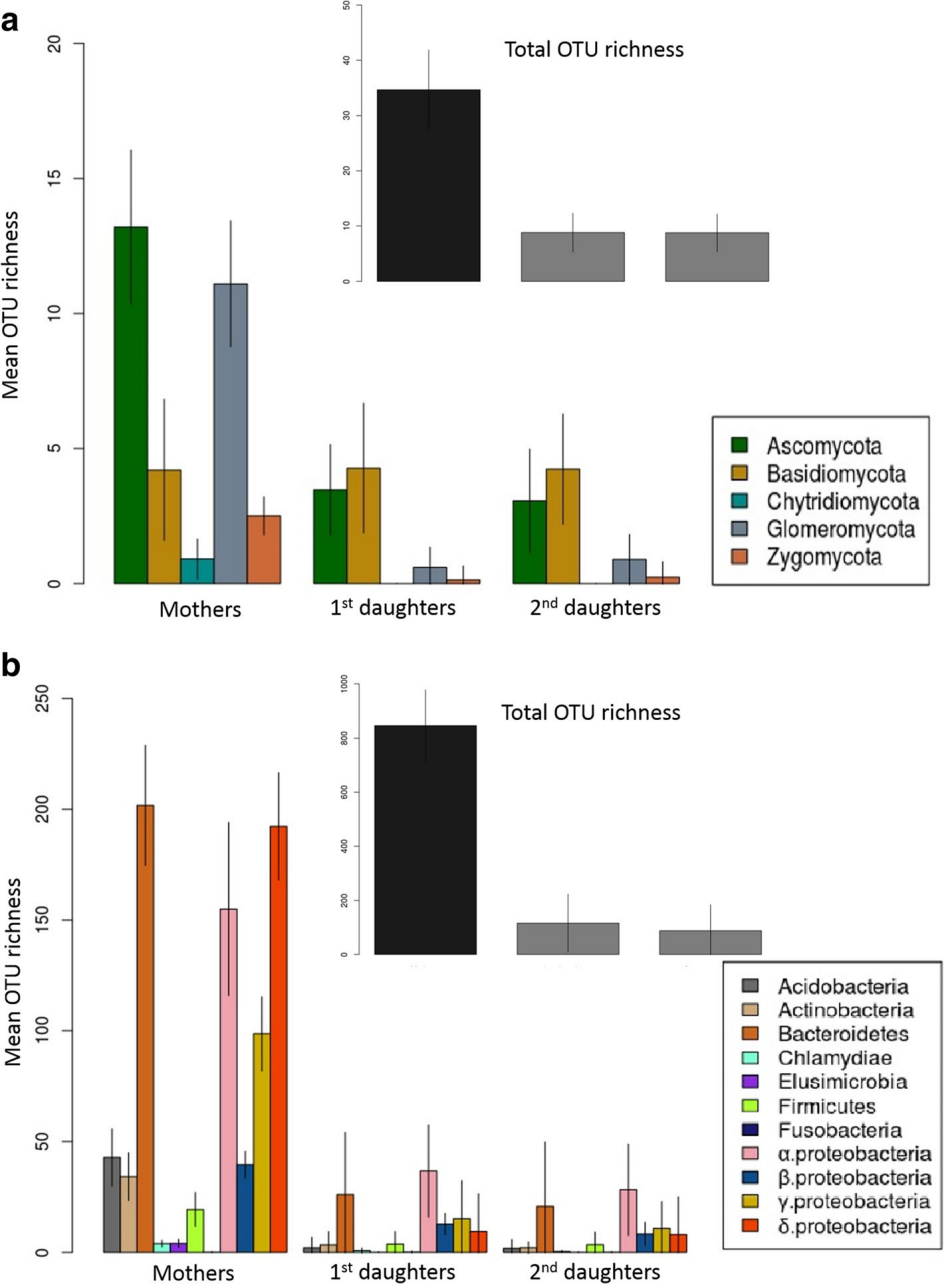
Composition of the bacterial and fungal communities within the root endosphere at the different positions in the clonal network. **a** Mean number of OTUs of each fungal phylum and mean total number of OTUs for all phyla together found in the root samples at the different positions in the clonal network (mother, 1st daughter, or 2nd daughter). Vertical bars represent the standard error of the mean for each phylum. The linear mixed models testing the differences in OTUs richness between mothers and daughters in the clonal network were significant *P* < 0.001. **b** Mean number of OTUs of each bacterial phylum and mean total number of OTUs for all phyla together found in the root samples at the different positions in the clonal network (mother, 1st daughter or 2nd daughter). Vertical bars represent the standard error of the mean for each phylum. The linear mixed models testing the differences in OTU richness between mothers and daughters in the clonal network were significant *P* < 0.001

Archaea (only *Thaumarcheota* phylum), fungi, and bacteria were found in mother ramets. Archaea were not detected in the daughter ramets, but fungi and bacteria were found in daughter roots (Fig. 2). Comparison of the sequences obtained from the roots of mother and daughter ramets revealed a subset of 100% identical reads in both mother and daughter ramets, representing 34 and 15% of the daughter fungal and bacterial reads respectively. Heritability, calculated as the number of OTUs found in the mother and in the roots of at least two daughters, varied from 15 to 374 OTUs (*μ* = 100.2 ± 118.6) for bacteria and from 0 to 12 OTUs (*μ* = 6.1 ± 3.63) for fungi, depending on the ecotype. To test whether this observed heritability was higher than would be expected stochastically (i.e., random dispersal of OTUs), we used a null model approach in which the identity of the fungi or bacteria species in the experimental samples was randomized while keeping the OTU richness identical. For each ecotype, we thus generated bacterial and fungal random daughter communities by sampling species from all the mother roots communities (regional pool) and compared the observed heritability in our dataset to this distribution of random heritability values. The null model approach indicated that the observed communities displayed significantly higher OTUs heritability between the roots of mothers and daughters than expected stochastically (one sample *t* test with alternative hypothesis “higher,” *P* < 0.01 *t* = 3.03, df = 8, and *P* < 0.001 *t* = 6.11, df = 9 for fungi and bacteria respectively) (Additional file 1: Figure S2). In addition to the non-random presence of OTUs in daughter roots we also found communities of fungi and bacteria in the stolon internodes connecting the ramets in the network (Additional file 1: Figure S3). These internodes exhibited similar phyla richness to that observed in the daughter roots. The transmission of bacteria and fungi within the *G. hederacea* clonal network was thus clearly demonstrated.

### Microbial communities filtration during transmission

Endophytic microorganisms were strongly filtered during the transmission process. Daughter roots displayed significantly lower fungal OTUs richness than mother ramet roots with mother communities averaging 40 OTUs compared to an average of 10 OTUs in the daughter ramets (linear mixed model, *F*_1,31_ = 280, *P* < 0.001; mother ramet 40 ± 7; daughter ramet 10 ± 3) (Fig. 2; Additional file 1: Table S1). The same significant pattern was observed for bacteria with mother communities averaging 800 OTUs compared to an average of 100 OTUs in the daughter ramets (linear mixed model, *F*_1,39_ = 410, *P* < 0.001; mother ramet 800 ± 131; daughter ramet 100 ± 100 Fig. 2, Additional file 1: Table S1). The observed “low” richness of the transmitted communities indicates that the transmitted microbiota is filtered from the original pool (i.e., the mother microbiota). A significant effect of ecotype, on the richness of the transmitted microbiota, was also found (see “Methods” section for details on the statistics and random factor used). Comparison of the microorganisms in the roots of mothers and daughters revealed a general decrease in richness of most phyla during the transmission process. The fungal communities colonizing the roots were mostly from the phyla *Ascomycota* (106 OTUs) and *Basidiomycota* (39 OTUs) and to a lesser extent from *Glomeromycota* (24 OTUs, recently suggested to be a sub-phylum Glomeromycotina [43]), *Zygomycota* (7 OTUs) and *Chytridiomycota* (4 OTUs) (Fig. 2a). The mean OTU richness of *Ascomycota* and *Glomeromycota* was significantly lower in daughter roots than in mother roots (Additional file 1: Table S1) whereas no significant variation was observed in the OTU richness of *Basidiomycota.* (Additional file 1: Table S1). This striking observation clearly advocates for the presence of a fungus-dependent filtering mechanism. The bacterial communities colonizing the roots were distributed in 3384 OTUs mostly belonging to *Proteobacteria* (2009 OTUs) and *Bacteroidetes* (715 OTUs) which together represented about 80% of all the sequences, the remaining 20% belonging to 6 additional phyla (Fig. 2b). Consistently with fungi, the bacterial OTU richness was significantly lower in daughter roots than in mother roots for the *Proteobacteria*, *Bacteroidetes*, *Acidobacteria*, *Actinobacteria*, and *Firmicutes* (Additional file 1: Table S1). This observation suggests that bacterial phyla are indifferently affected by the filtering mechanism.

### The heritability of a specific cohort of microorganisms

**Fig. 3:**
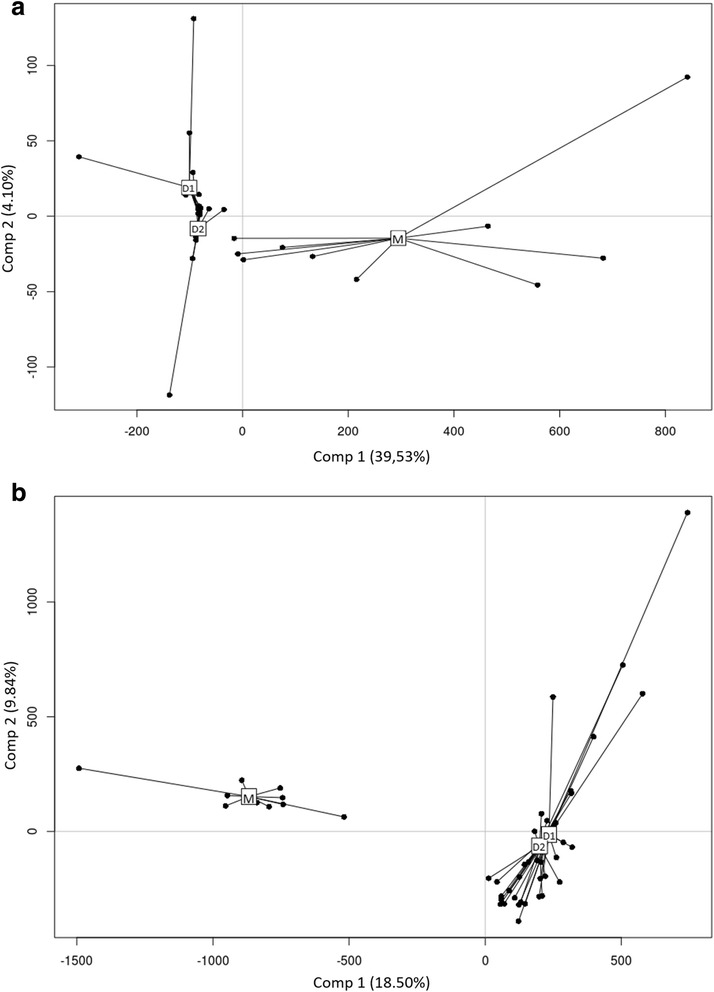
Partial least square discriminant analysis (PLS-DA). **a** PLS-DA testing the significance of the position (mothers, 1st daughters, and 2nd daughters) on the composition of the root bacterial communities. **b** PLS-DA testing the significance of the position (mothers, 1st daughters, and 2nd daughters) on the composition of the root fungal communities. The groups used as grouping factor in the model are represented on the graphs. They correspond to mother, 1st and 2nd daughter ramets. 1st and 2nd ramets were grouped independently of the stolon to which they belonged. This analysis was used to test the hypothesis that roots at different ramet positions in the clonal network exhibit similar compositions of fungal and bacterial communities. The percentage of variance indicated on each axis represents the variance of the communities composition explained by the grouping factor

The differences in microorganism community composition between mother and daughter roots were assessed using a multi-regression approach with a partial least squares discriminant analysis procedure (PLS-DA) (see Material and Methods, Additional file 1). The advantage of this analysis is its ability to test a hypothesis based on a grouping factor of the samples in the dataset (i.e., an explicative factor) and to obtain the significance of the factor as well as the part of the variance explained by the factor. With this analysis, the entire dataset can be used and most of the variance conserved in contrast to NMDS approaches in which the distances between samples such as Bray-Curtis or Jaccard summary the variance between samples. Significant differences in the composition of daughters communities compared to mothers’ were detected for both fungi (*P*_PLS-DA_ = 0.001, *P*_Mothers vs Daughters_ < 0.01, explained variance = 87.3%, Fig. 3a) and bacteria (*P*_PLS-DA_ = 0.001, *P*_Mothers vs Daughters_ < 0.01, explained variance = 72.4%, Fig. 3b; Additional file 1: Table S2). These differences in composition between mothers and daughters can be explained by the observed diminution in richness during the transmission process. These results indicate that only a portion of the original pool of microorganisms is transmitted from the mother to the daughters (i.e., a specific set of organisms). To test the hypothesis of a plant filtering conducting to the transmission of a specific cohort of microorganisms we compared the microbiota composition within the daughter roots using a PLS-DA procedure. The composition of the roots communities was not significantly different between the 1st and 2nd daughter ramets (*P*_PLS-DA_ = 0.09 and *P*_PLS-DA_ = 0.33 for fungi and bacteria respectively (Additional file 1: Table S2), thus confirming that a specific set of organisms was similarly transmitted to daughter-plants of all ecotypes.

### Effect of dispersal distance and dispersal time

We found patterns of richness dilution in bacterial communities along the stolons (linear mixed model, *F*_1,18_ = 6.13, *P* < 0.05, Additional file 1: Table S3) showing that those ramets most distant from the mother were less rich in bacteria than the closer ramets. This finding suggests that colonization of the daughters by bacteria is limited by dispersal distance. This pattern of richness dilution also followed the course of plant development as stolons produced earlier in the experiment (i.e., 1st stolon emitted by the plant) were found to be richer (linear mixed model, *F*_1,9_ = 4.92, *P* < 0.05, Additional file 1: Table S3), which suggests that richness of the bacterial community also depends on dispersal time. Alternatively, these patterns may be linked to a cumulative filtering effect at each node of the clonal network, reducing the pool of transmitted bacteria. Conversely, these richness dilution patterns were not detected for fungal communities (Additional file 1: Table S1), suggesting either that dispersion of the transmitted species was not limited or that the fungal community was already strongly filtered during the initial transmission. These two non-exclusive hypotheses are supported by our observation of a variation in the diminution of fungal community richness between mothers and daughters, probably dependent on the life history and dispersal traits of the different fungal taxonomic groups.

## Discussion

This work provides the first demonstration of vertical transmission and heritability of a specific endospheric microbiota (fungi and bacteria) in plants. Our work echoes with previous work demonstrating the transmission of microorganisms between plants through common mycelial networks [44–46]. However, the transmission of microorganisms through hyphal network is different from the clonal network in the way that such networks are not constituted of plant tissues and thus the environmental filters occurring on microorganisms (i.e., selection pressures) are not the same. In the case of clonal plants, the immune system of the plant should apply a strong selective pressure on the transmitted microorganisms. Along with other studies, it supports an understanding of the plant as a complex—rather than a standalone—entity and is aligned with the idea that the plant and its microbiota have to be considered as holobionts [5, 8, 47]. Our demonstration of microbiota transmission supports the idea that microbial consortia and their host constitute a combined unit of selection. This finding does not conflict with the idea that this heritability of microbiota (microbial components metaphorically called “singers” in Doolittle and Booth 2017) [48], within clonal plants, in fact consists of the heritability of a selected set of functions (the “song” in Doolittle and Booth, 2017) [47]. Our work thus highlights evolutionary processes at work within the holobiont entity and reconciles holobiont and evolutionary approaches of the on-going debate [47, 49, 50].

For the plant, the transmission of a microbiota along plant clonal networks extends to microorganisms the concept of physiological integration previously demonstrated for information and resources. This integrated network-architecture questions the idea of a meta-holobiont organization where ramets (i.e., holobionts) can act as sinks or sources of micro-organisms. Such a structure may ensure exchanges between the holobionts, and especially between the mother source and the daughter “sinks,” thereby increasing the fitness of the clone as a whole. Indeed, the inheritance of a cohort of microorganisms that has already gone through the plant filtering system provides a pool of microorganisms available for recruitment in the newly colonized environments. This “toolbox” of microorganisms could allow the plant to rapidly adjust to environmental conditions and therefore provide fitness benefits in a heterogeneous environment [19]. This may be assimilated to plant niche construction and provide a competitive advantage when colonizing new habitats.

From the perspective of microorganisms, the stolons can be seen as ecological corridors facilitating the dispersal at a fine scale. In addition to propagules transport in the environment, this process ensures a spread of the transmitted organisms from one suitable host to another. As a consequence, transmitted symbiotic partners may benefit from a priority effect when colonizing the rooting system within the new environment [51]. Future work will thus need to address (i) the direction (uni vs. bidirectional) of microorganisms transmission within the clonal network as well as the modalities of (ii) the transmission mechanism (active or passive), and of (iii) microorganisms filtering during this transmission to determine (iv) the significance of the process in ecosystems. As regards this last aspect, plant communities are dominated by clonal plants and our findings demonstrate their fundamental role in the spreading of microorganisms between trophic levels and reveal a new ecological function of plant clonality. Considering that the heritability process demonstrated herein affects different compartments within the ecosystem, this novel ecosystem process consisting of microbiota filtering and transfer by clonal plants is of paramount importance.

## Conclusion

The results presented herein demonstrated the transmission of a part of the microbiota of the clonal plant *Glechoma hederacea* to its clonal progeny. We evidenced that only few specific microorganisms were transmitted, suggesting the existence of a filtering process during the transmission. These findings demonstrate the transmission of a specific cohort of microorganisms between clonal generation and impact our understanding of the plant holobiont. In the context of clonal plants, different holobionts are connected within a common network were microorganisms can be exchanged, constituting another level of organization of the holobiont for clonal organisms.

## Additional files

Additional file 1: **Figures S1-S3** and **Tables S1-S3**. Additional results and material and methods information. (DOC 1670 kb)

Additional file 2: Archaeal dataset. (XLS 34 kb)

Additional file 3: Bacterial dataset. (XLS 2420 kb)

Additional file 4: Fungal dataset. (XLS 166 kb)
