# Supplementary material for: A microorganisms’ journey between plant generations
Source: Microbiome. 2018 Apr 26;6:79. doi: 10.1186/s40168-018-0459-7 (PMC5918900; doi:10.1186/s40168-018-0459-7)
Supplement: Supplementary file 1 — Figures S1-S3 and Tables S1-S3. Additional results and material and methods information. (DOC 1670 kb) [file 40168_2018_459_MOESM1_ESM.doc]

*******

**ADDITIONAL FILE 1**

***

A microorganisms journey between plant generations

**Nathan Vannier^1^, Cendrine Mony^1^, Anne-Kristel Bittebiere^2^, Sophie Michon-Coudouel^1^, Marine Biget^1^, Philippe Vandenkoornhuyse^1*^**

^1^ Université de Rennes 1, CNRS, UMR 6553 EcoBio, campus Beaulieu, Avenue du Général Leclerc, 35042 RENNES Cedex (France)

^2^ Université de Lyon 1, CNRS, UMR 5023 LEHNA 43 Boulevard du 11 Novembre 1918, 69622 VILLEURBANNE Cedex (France)

*Corresponding author. Email: philippe.vandenkoornhuyse@univ-rennes1.fr

**Table S1 | Results of linear mixed models comparing OTU richness of mother roots to daughter roots for all fungi and all bacteria as well as for specific phyla.** P-values and F-values are presented as well as degrees of freedom.

| Mothers vs Daughters |  |  |  |
| --- | --- | --- | --- |
| Fungi | num DF/ den DF | F-value | P-value (α=0.05) |
| All fungi | 1/31 | 280 | <0.001 |
| Ascomycota | 1/31 | 177 | <0.001 |
| Basidiomycota | 1/31 | 0.07 | 0.79 |
| Glomeromycota | 1/31 | 460 | <0.001 |
| Bacteria | num DF/ den DF | F-value | P-value (α=0.05) |
| All bacteria | 1/39 | 410 | <0.001 |
| Acidobacteria | 1/39 | 509 | <0.001 |
| Actinobacteria | 1/39 | 241 | <0.001 |
| Bacteroidetes | 1/39 | 293 | <0.001 |
| Firmicutes | 1/39 | 73 | <0.001 |
| Proteobacteria | 1/39 | 402 | <0.001 |

**Table S2 | PLS-DA (Partial Least Square Discriminant Analysis) results of the different models tested for different grouping of ramets position in the clonal network.** The significance of the model is indicated by a P-value. When the model is statistically significant, the statistical sensitivity is indicated by the number and percentage (in brackets) of misclassifications of samples in categories accepted by the class model. The modelling efficiency is also presented in the form of the percentage of variance explained by the model. When the model is significant, the P-values of the pairwise test comparison of the different groups are presented. M = mother, D1 = 1^st^ daughter, D2 = 2^nd^ daughter, D1-1 = 1^st^ daughter on the first stolon, D1-2 = 1^st^ daughter on the second stolon, D2-1 = 2^nd^ daughter on the second stolon, D2-2 = 2^nd^ daughter on the second stolon.

| M vs D1 vs D2 | P-value | Misclassifications (%) | Explained Variance % | P-values pairwise tests |
| --- | --- | --- | --- | --- |
| Fungi | 0.002 | 37.9 (1.3) | 43.62 | A-B=0.0015, A-C=0.0015, B-C=0.084 |
| Bacteria | 0.001 | 43.8 (1.7) | 28.34 | A-B=0.0015, A-C=0.0015, B-C=0.46 |
| M vs D1D2 |  |  |  |  |
| Fungi | 0.001 | 10.7 (1.1) | 87.31 | ______ |
| Bacteria | 0.001 | 0.2 (0.2) | 72.4 | ______ |
| D1 vs D2 |  |  |  |  |
| Fungi | 0.091 | ______ | ______ | ______ |
| Bacteria | 0.333 | ______ | ______ | ______ |
| D1-1 vs D1-2 vs D2-1 vs D2-2 |  |  |  |  |
| Fungi | 0.568 | ______ | ______ | ______ |
| Bacteria | 0.162 | ______ | ______ | ______ |

**Table S3 |** Results of linear mixed models comparing OTU richness between daughter roots depending on their position in the network (1^st^ or 2^nd^ daughter, 1^st^ or 2^nd^ stolon) for all fungi and all bacteria. P-values and F-values are presented as well as degrees of freedom.

| 1^st^ Daughters vs 2^nd^ Daughters | num DF/den DF | F-value | P-value (α=0.05) |
| --- | --- | --- | --- |
| All fungi | 1/11 | 0.001 | 0.97 |
| All bacteria | 1/18 | 6.13 | 0.03 |
| Stolon (1^st^, 2^nd^) | num DF/den DF | F-value | P-value (α=0.05) |
| All fungi | 1/8 | 0.02 | 0.88 |
| All bacteria | 1/9 | 4.92 | 0.04 |


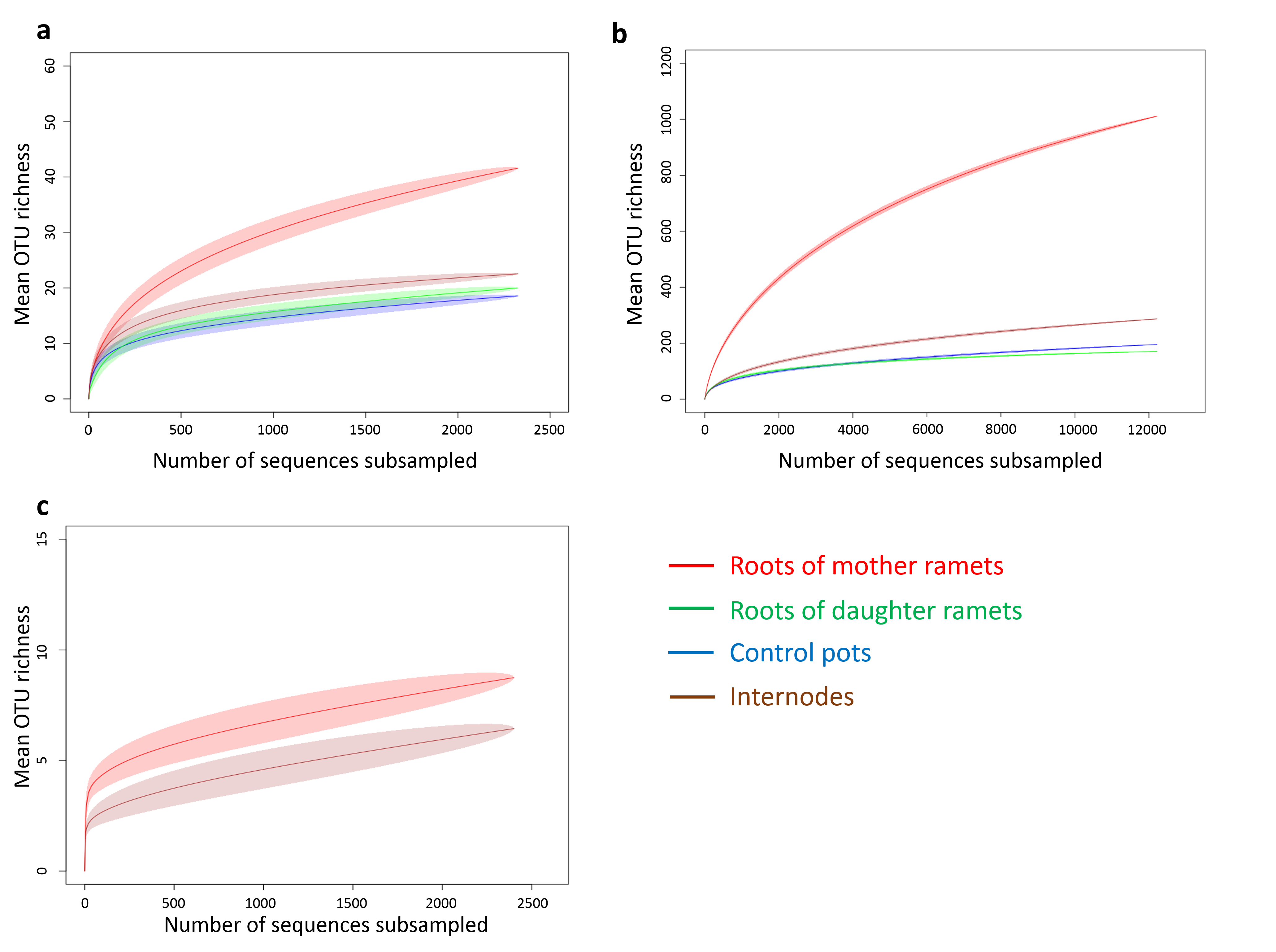


**Figure S1 |** (**a**), mean rarefaction curves for the bacterial communities in the mother roots samples (red), the daughter roots samples (green), the internode samples (brown) and the control pots (blue). (**b**), mean rarefaction curves for the fungal communities in the mother roots samples (red), the daughter roots samples (green), the internode samples (brown) and the control pots (blue). (**c**), mean rarefaction curves for the fungal communities in the mother roots samples (red), the daughter roots samples (green), the internode samples (brown) and the control pots (blue). Coloured ideas indicate ± SE.


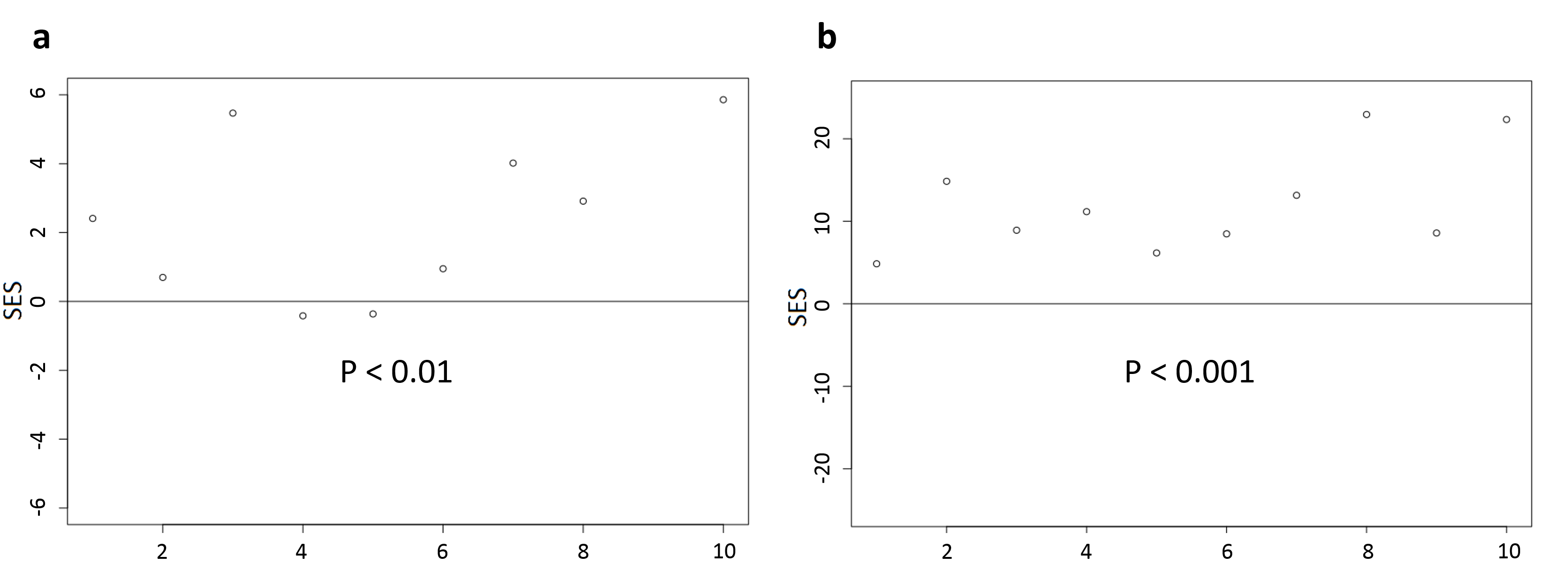


**Figure S2 | Null model results.** (**a**), Standard Effect Size (SES) values for fungal root communities of the different ecotypes. (**b**), Standard Effect Size (SES) values for bacterial root communities of the different ecotypes. One value in the graph corresponds to one ecotype. To determine whether the observed heritability could be expected stochastically, we compared the observed heritability against a null model. We built a null model for each daughter ramet of the different ecotypes (9 ecotypes for fungi and 10 for bacteria) by generating daughter communities with random samples of the microorganisms species occurring within the species pool (regional pool) of the mother ramet. Only the species identity was changed while species richness within the null daughter communities remained unmodified. We created 9999 null datasets for each daughter ramet and measured the OTUs heritability for each ecotype created in this way as the number of OTUs shared between the mother and at least 2 daughters. We then calculated the Standard effect size (SES) values of each ecotype. Negative SES values indicated that the observed heritability was lower than would be expected in the null-model (heritability of OTUs not specific to the ecotype mother ramet), whereas positive SES values revealed a higher heritability than expected (heritability of microorganisms from the mother). The horizontal line represents an SES value of 0 (no difference between the observed heritability and the null heritability). P-values indicate the significance of the one sample t-test to determine whether SES values are significantly higher than 0 (alternative hypothesis “greater”).


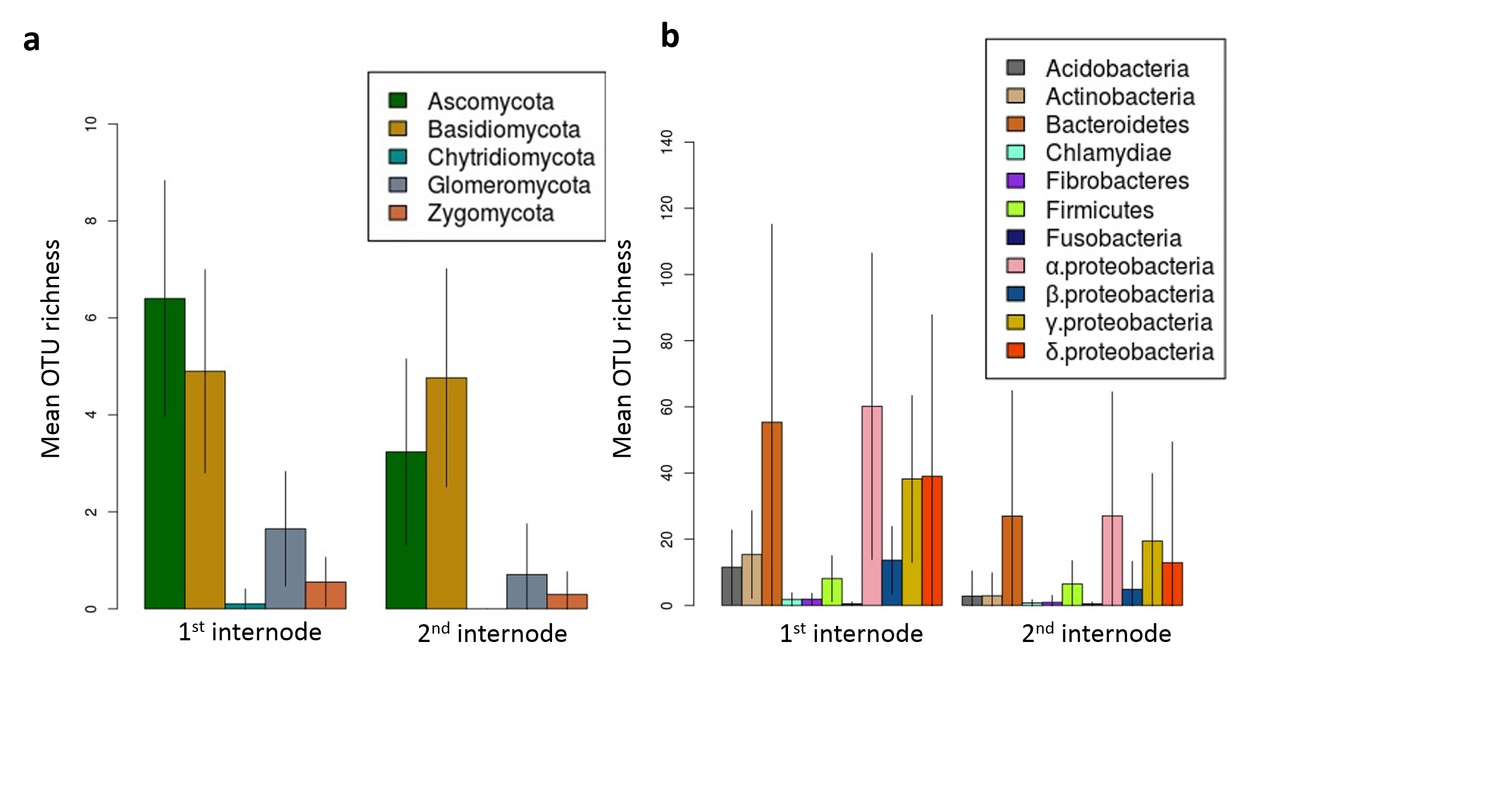


**Figure S3 | Composition of the bacterial and fungal communities within the internode samples.** (**a**), mean number of OTUs of each fungal phylum found in the internode samples at the different positions in the clonal network (1^st^ internodes following the mother ramets and 2^nd^ internodes following the 1^st^ daughter ramets). Vertical bars represent the standard error of the mean for each phylum. (**b**), mean number of OTUs of each bacterial group (phylum or class) found in the internode samples at the different positions in the clonal network (1^st^ internodes following the mother ramets and 2^nd^ internodes following the 1^st^ daughter ramets). Vertical bars represent the standard error of the mean for each phylum.

**Supplementary material and method | Composition of the watering solution**

The watering solution contained KNO_3_ (0.6066 g.L^-1^), Ca(NO_3_)_2_.4H_2_O (0.9446 g.L^-1^ ), NH_4_NO_3_ (0.0800 g.L^-1^) , NH_4_H_2_PO_4_ (0.0575 g.L^-1^), MgSO4.6H2O (0.0228 g.L^-1^), KCl (0.0037 g.L^-1^), H_3_BO_3_ (0.0015 g.L^-1^), MnSO_4_.4H_2_O (0.0005 g.L^-1^), ZnSO_4_.7H_2_O (0.0006 g.L^-1^), CuSO_4_.5H_2_O (0.0001 g.L^-1^)
